# Transcriptional and Metabolic Networks Underlying Melanin Deposition in Silkie Chicken Muscle: A Multi-Omics Insights

**DOI:** 10.3390/ani16020252

**Published:** 2026-01-14

**Authors:** Yuxian Pan, Lin Zhang, Xin Yue, Zhen Sun, Huaiyong Zhang, Xuemeng Si, Rui Zheng, Wen Chen, Meng Zhang, Yanqun Huang

**Affiliations:** 1 College of Animal Science and Technology, Henan Agricultural University, Zhengzhou 450046, China; panyuxian2000@163.com (Y.P.); zhanglin9916@outlook.com (L.Z.); yuexindr@outlook.com (X.Y.); zhensun87328@163.com (Z.S.); huaiyzhang@outlook.com (H.Z.); sxmswun@126.com (X.S.); ruizheng1212@126.com (R.Z.); cchen.wen@aliyun.com (W.C.); 2 Annoroad Gene Technology (Beijing) Co., Ltd., Beijing 100176, China

**Keywords:** silkie chicken, melanin deposition, pectoralis muscle, transcriptomics, metabolomics

## Abstract

Silkie (SK) chickens, valued for dark meat, serve as a model to study melanin deposition in muscle. Integrated transcriptomics and metabolomics of SK vs. Arbor Acres (AA) broiler pectoralis were used to identify key molecular drivers of meat color. All birds were cage-raised under standardized temperature and light conditions with free access to feed and water. Pectoralis muscle samples were collected from 24-day-old healthy SK and AA chickens (*n* = 6). Transcriptome profiling identified 488 differentially expressed genes in SK chickens, with seven conserved melanogenesis genes (*TYRP1*, *MLANA*, *TYR*, *MLPH*, *EDNRB2*, *PMEL*, *GPNMB*) consistently upregulated across dark-pectoralis breeds, and melanogenesis and WNT pathways were activated. Co-expression network analysis highlighted *SOX10* as a key hub regulator. Metabolomics quantified 129 differentially abundant metabolites. A critical finding was the significant depletion of L-tyrosine and its derivatives in SK muscle, despite upregulated melanogenesis genes. It indicates intense metabolic flux toward pigment synthesis. Integrated analyses converged on tyrosine metabolism and redox pathways: oxidized glutathione and p-coumaric acid correlated negatively with pigment deposition, while ADP-ribose and pyridoxal correlated positively. Additionally, novel inhibitors *PNMT* and *HIBADH* may modulate melanin deposition. These findings reveal a trade-off between pigment deposition and redox balance, providing molecular markers for poultry melanin-related trait improvement.

## Simple Summary

Silkie chickens exhibit ectopic muscle melanin, with transcriptomics identifying 488 differentially expressed genes and seven conserved melanogenesis genes upregulated. Key pathways include melanogenesis and WNT signaling pathways; *SOX10* is a hub regulator. Metabolomics revealed depleted L-tyrosine and derivatives, indicating active pigment synthesis. Integrated analysis linked tyrosine metabolism and redox balance, with metabolites like glutathione and p-coumaric acid negatively correlated, and ADP-ribose and pyridoxal positively associated. Novel inhibitors *PNMT* and *HIBADH* may modulate melanin deposition. Findings highlight a trade-off between pigmentation and redox homeostasis, offering molecular insights for trait improvement.

## 1. Introduction

In black-boned chickens, melanin deposition in the muscle is a noteworthy phenomenon. The Silkie (SK) chicken, a celebrated Chinese breed valued for its dual medicinal and culinary significance, stands out distinctly from conventional poultry due to its unique “five-black” phenotype that encompasses black meat, bones, skin, comb, and viscera [1]. This unique trait, characterized by extensive pigmentation in tissues including skin, muscles, and visceral organs [2,3], not only serves as a distinctive phenotype marker but also confers significant functional advantages. Notably, the dark pigmentation in Silkie pectoralis muscle is associated with enhanced antioxidant capacity [4], superior meat quality [5], and potential health benefits ranging from immunomodulatory properties [6,7,8] to metabolic regulation [9]. These characteristics have sparked considerable scientific interest and commercial value, driving efforts to unravel the molecular mechanisms underlying muscle melanogenesis.

In contrast, Arbor Acres (AA) chicken, a globally widely used commercial broiler breed, exhibits a stable phenotype with no melanin deposition in muscle tissues [10,11]. With publicly available genome information and a well-characterized genetic background, AA broilers are commonly employed as a phenotypic control in studies exploring the mechanisms of phenotypic variation in poultry. Their extreme divergence from SK chickens in muscle pigmentation provides a robust comparative framework for identifying core regulators and pathways associated with ectopic melanin deposition in muscle.

The biochemical pathway of melanin synthesis is well-characterized in cutaneous tissues, involving the tyrosinase-mediated conversion of tyrosine to dopaquinone and the subsequent formation of eumelanin and pheomelanin [12,13,14,15]. Key regulators such as *MITF* and its downstream targets (*TYR*, *TYRP1* and *DCT*) have been extensively studied in skin melanocytes [7,16]. However, the mechanisms governing melanin deposition in skeletal muscle remain poorly understood, particularly regarding tissue-specific regulation and the interplay between genetic and metabolic factors. Recent transcriptomic studies in various black-boned chicken breeds have identified candidate genes including *EDNRB2*, *GPNMB*, and *TRPM1* [17,18,19,20], while metabolomic analyses have highlighted the importance of tyrosine and glutamic acid [21]. Notably, the activity of tyrosinase directly determines the rate of melanin synthesis [22,23]. Glutamic acid, as a precursor for glutathione synthesis, is intricately linked to the precise regulation of eumelanin/pheomelanin ratios by glutathione [24]. Despite these advances, a comprehensive understanding of the gene-metabolite networks regulating muscle melanogenesis is still lacking.

To address this knowledge gap and gain a holistic understanding of the distinct dark meat phenotype in SK chickens, this study employed an integrated multi-omics approach that combined transcriptome and broad-targeted metabolome profiling of pectoralis muscle from SK and AA broilers, leveraging their extreme phenotypic divergence. It aimed to systematically identify differentially expressed genes (DEGs) and differentially abundant metabolites (DAMs), delineate significantly enriched biological pathways, and construct gene-metabolite interaction networks that collectively regulate melanin deposition in this tissue. By maximizing the capture of key regulatory factors through this extreme phenotypic comparison, we identified the core gene-metabolite network underlying muscle melanin deposition, thereby providing candidate targets for subsequent validation studies within a uniform genetic background.

## 2. Materials and Methods

### 2.1. Animal Samples and Collection

A total of 240 one-day-old male chickens, including 120 SK chickens and 120 AA broilers (provided by Kaifeng Xingda Poultry Co., Ltd., Kaifeng, China), were raised in cages within a temperature- and light-controlled room (34 °C gradually reduced to 22 °C by day 24; 23 h light: 1 h dark for the first 7 days, then 18 h light: 6 h dark) until 24 days of age. All birds had free access to feed and water, with diets formulated according to the Chinese Standard for Chicken Feeding (NY/T 33-2004) [25]. Six healthy SK chickens and six AA broilers, with close to population average body weights (800 ± 50 g for AA and 330 ± 30 g for SK), respectively, were randomly selected from two populations at 24 days old. Birds were sacrificed at 24 d, and pectoralis muscle samples were immediately flash-frozen in liquid nitrogen and stored at −80 °C for subsequent transcriptomic and metabolomic analyses. The animal experimental protocols were duly authorized by the Institutional Animal Care and Use Committee (IACUC) of Henan Agricultural University (approval number: HNND-2021-104, approval date: 15 September 2021).

### 2.2. RNA Extraction, cDNA Synthesis, and Real-Time Quantitative PCR (qRT-PCR)

Total RNA was extracted from pectoralis muscle tissue using Trizol RNA isolation reagent (Invitrogen, Carlsbad, CA, USA). The concentration and quality of the isolated RNA were determined using a spectrophotometer (Thermo NanoDrop One, Waltham, MA, USA) and agarose gel electrophoresis. The OD 260/OD 280 ratio of all samples ranged from 1.8 to 2.0. For first-strand cDNA synthesis, 500 ng of total RNA was reverse-transcribed into double-stranded cDNA in a 20 μL reaction mixture employing the HiScript II 1st Strand cDNA Synthesis Kit (including gDNA wiper) (Vazyme Biotech Co., Ltd., Nanjing, China). The qRT-PCR primers were designed using the NCBI Primer-BLAST online tool (https://www.ncbi.nlm.nih.gov/tools/primer-blast/, accessed on 12 April 2025), and the primer sequences are listed in Supplementary Table S1. The obtained cDNA was used for qRT-PCR on a LightCycler 96 instrument (BioRad, Hercules, CA, USA) with SYBR Green qRT-PCR master mix (Vazyme Biotech Co., Ltd., Nanjing, China). The 10 μL qPCR reaction mixture consisted of 5 μL of 2× SYBR Green Premix Ex Taq™ (Vazyme Biotech Co., Ltd., Nanjing, China), 0.2 μL (0.1 μM each) of forward and reverse primers, 1 μL of cDNA template, and 3.6 μL of nuclease-free water. The PCR amplification protocol was set as follows: initial denaturation at 95 °C for 30 s, followed by 35 cycles of 95 °C for 10 s and 60 °C for 30 s. The relative abundance of target genes was analyzed using the 2^−ΔΔCt^ method and normalized to *β-actin* [26]. Each group contained at least three biological replicates, with each biological replicate comprising three technical replicates.

### 2.3. Transcriptome Profiling in the Pectoralis Muscle

5 μg of high-quality RNA from each sample (*n* = 6 for SK and AA birds, respectively) was processed following the strand-specific RNA sequencing (ssRNA-Seq) method. Ribosomal RNA was first depleted using the Epicentre Ribo-zero™ Kit (Epicentre, Madison, WI, USA), followed by library construction with the NEBNext Ultra™ Directional RNA Library Prep Kit (NEB, Ipswich, MA, USA). The detailed workflow comprised the following steps: first-strand cDNA synthesis using random hexamer primers and RNaseH-deficient M-MuLV reverse transcriptase. Second-strand cDNA synthesis employing DNA polymerase I and RNase H, with dUTP substitution for dTTP in the reaction buffer; purification of the resulting products using the AMPure XP system, with subsequent quality assessment performed on an Agilent 2100 Bioanalyzer. After library construction, quantification was performed using Qubit2.0, and the insert size was detected using Agilent 2100 (Agilent Technologies, Santa Clara, CA, USA). Qualified libraries were pooled and subjected to HiSeq sequencing (Novogene, Beijing, China). Subsequently, raw sequencing data were processed using Fastp (v0.19.3) to remove adapter-containing reads, low-quality sequences, and those with a proportion of unknown bases (N) greater than 10%, resulting in high-quality clean reads for downstream analysis. Next, HISAT2 v2.0.5 software was utilized to align the clean reads to the chicken reference genome (http://ftp.ensembl.org/pub/release-105/fasta/gallus_gallus/dna/, accessed on 2 February 2025, GRCG6a). Assembly of the mapped reads from each sample was performed via StringTie (v1.3.3b) following a reference-based strategy. Meanwhile, Feature Counts (1.5.0-p3) served to quantify the reads mapped to each gene, and calculations of the Fragments Per Kilobase Million (FPKM) values for each gene were based on gene length and mapped reads.

To systematically characterize transcriptional signatures associated with melanogenesis, we performed integrated transcriptomic analyses using the following workflow: Orthogonal Partial Least Squares-Discriminant Analysis (OPLS-DA) was employed to visualize inter-group differences via Metware cloud (https://cloud.metware.cn/, accessed on 12 February 2025), while a correlation coefficient matrix was used to evaluate sample-to-sample consistency using bioinformatics platform (https://www.bioinformatics.com.cn/, accessed on 15 February 2025). DEGs between SK and AA birds were identified using stringent thresholds (|log2FC| ≥ 1, FDR < 0.05; Metware Cloud). Genes with log2FC > 3 and FDR < 0.05 (SK vs. AA) were classified as highly expressed in SK birds. To further explore the identified DEGs, we leveraged a web-based bioinformatics platform for various analyses. This included generating visualizations such as the volcano plot and cluster heatmap, as well as performing functional annotation via Gene Ontology (GO) enrichment analysis and Kyoto Encyclopedia of Genes and Genomes (KEGG) pathway enrichment analysis.

For the co-expression analysis, to identify high-quality candidate genes influencing melanin deposition, key melanogenesis regulators were first pinpointed through a multi-step process. This involved using Venn analysis to screen for common DEGs from the transcriptome sequencing of pectoralis muscle tissues in the SK vs. AA comparison, cross-referencing these DEGs with data from three additional transcriptome sequencing studies. These studies compared dark-breasted and light-breasted chickens, namely Wuliangshan black-boned chicken (WLS) vs. Cobb broiler (CB) [19], WLS vs. Chahua chicken (CH) [19], and hyperpigmented Xuefeng black-bone chicken (XFH) vs. hypopigmented Xuefeng black-bone chicken (XFL) [18]. Additionally, several highly expressed genes (in SK) with experimentally validated functions in melanin deposition were selected as key melanogenesis genes. Ultimately, 13 genes were identified as key melanogenesis regulators.

Co-expression analysis was then conducted between these 13 regulators and the DEGs from the SK vs. AA comparison, using the Pearson correlation method (with a correlation coefficient |*r*| > 0.9 and *p* < 0.05). The prioritized DEGs from this analysis were further processed using Cytoscape software (version 3.9.0, https://cytoscape.org, accessed on 25 February 2025) to construct a visualized interaction network. Finally, GO and KEGG functional enrichment analyses were performed on these genes via bioinformatics online tools. This multi-study integrative approach leveraged convergent genetic evidence across divergent avian models, establishing a robust framework to pinpoint melanogenic regulators in Silkie pectoralis muscle.

### 2.4. Widely Targeted Metabolomic Analyses on Pectoralis Muscle of Chickens

Global metabolite profiles were analyzed in the pectoralis muscle tissues of the same chickens used for ssRNA-Seq analysis, following the method described by Zhang et al. [27,28]. Briefly, 1 mL of 70% methanol was added to the homogenized sample and vortexed, and then centrifuged at 12,000 rpm for 10 min at 4 °C; after centrifugation, 400 µL of the supernatant was transferred to the corresponding Eppendorf tube, and the samples were stored at −20 °C overnight. The mixture was centrifuged again for 3 min (12,000 rpm, 4 °C), and the supernatant was collected for metabolomics analysis using ultra-performance liquid chromatography-tandem mass spectrometry (UPLC-MS/MS). Quality control (QC) samples were prepared by mixing equal volumes of extracts from all samples within the same group. The LC-ESI-MS/MS system (UPLC, ExionLC AD https://sciex.com.cn/, accessed on 3 February 2025; MS, QTRAP system https://sciex.com/, accessed on 3 February 2025) was used for the analysis of the extracted samples. All data analysis was based on the in-house Metware database (Metware Biotechnology Co., Ltd., Wuhan, China).

To further characterize the overall features of the metabolites, metabolomics data were analyzed using the online Metware software (version 14.0.0, https://cloud.metware.cn/, accessed on 22 March 2025). This included OPLS-DA to distinguish group differences and a correlation matrix to assess metabolite correlations. DAMs were identified based on the Variable Importance in Projection score (VIP) > 1 and |log2FC| ≥ 1 between SK and AA birds. The distribution of DAMs was visualized with a volcano plot. Hierarchical cluster analysis was performed on all DAMs, classifying them by primary category. KEGG pathway enrichment analysis was performed using Metware to explore their functional pathways.

### 2.5. Integrated Analysis of Pectoralis Muscle Transcriptomics and Metabolomics

To dissect the interplay between genes and metabolites, Metware was used for KEGG co-enrichment analysis of DEGs and DAMs from SK and AA chickens, aiming to identify shared enriched pathways. Top transcriptomic pathways (ranked by *p*-value) were visualized. An interaction network was built in Cytoscape (v3.9.0) to link DAMs and melanogenic genes with high Pearson correlations (|*r*| > 0.9, *p* < 0.05), revealing direct metabolic regulation of pigmentation.

### 2.6. Statistical Analysis

Statistical analyses for qRT-PCR were carried out using the statistical package SPSS 21.0 (SPSS Inc., Chicago, IL, USA). Differences between groups were tested using *t*-test for independent samples. Significant differences were defined as *p* < 0.05, and the data are presented as the mean ± standard error of the mean (SEM). Graphical plots were generated using GraphPad Prism 8 (GraphPad Software, San Diego, CA, USA).

## 3. Results

### 3.1. Transcriptome Profiling in the Pectoralis Muscle

**Figure 1:**
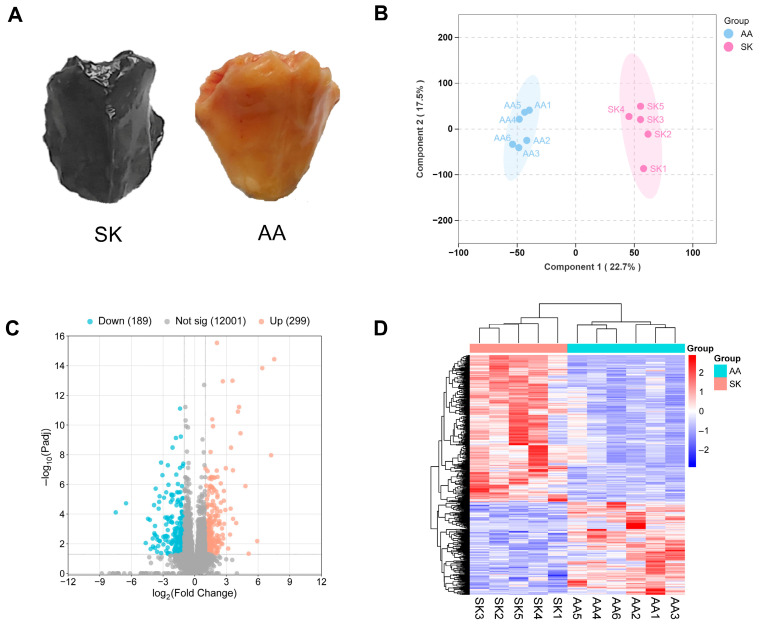
Identification of differentially expressed genes (DEGs) based on pectoralis muscle transcriptomics of SK vs. AA. (**A**) Great difference in color of pectoralis muscle between SK and AA chickens. (**B**) OPLS-DA analysis (the ellipse represents the 95% confidence intervals of the samples in each group), (**C**) Volcano plot and (**D**) Heatmap of DEGs in SK vs. AA comparison. Genes with |log2FC| ≥ 1 and FDR < 0.05 were considered as DEGs. SK: Silkie chickens; AA: Arbor Acres broilers.

The pectoralis muscle of SK chickens exhibited a significantly darker color compared to that of AA chickens (Figure 1A). To identify candidate genes underlying this divergence, we performed transcriptome sequencing on pectoralis muscle samples from both breeds. After quality control, all libraries except SK6 showed stable GC content and low base error rates (Supplementary Table S2). The SK6 sample was excluded from downstream analysis due to poor data quality. OPLS-DA confirmed distinct clustering between SK and AA birds (Figure 1B), while inter-sample correlation analysis (|*r*| > 0.9 across replicates; Supplementary Figure S1A) validated the reproducibility of the dataset. Differential expression analysis identified 488 DEGs including 299 upregulated and 189 downregulated in SK vs. AA. (Figure 1C and Supplementary Table S3). The reliability of RNA-seq results was further verified by qRT-PCR on ten randomly selected genes (Supplementary Figure S2). Hierarchical clustering of DEGs revealed breed-specific expression patterns (Figure 1D), underscoring the robustness of the transcriptomic approach to identify genes associated with meat color variation.

**Figure 2:**
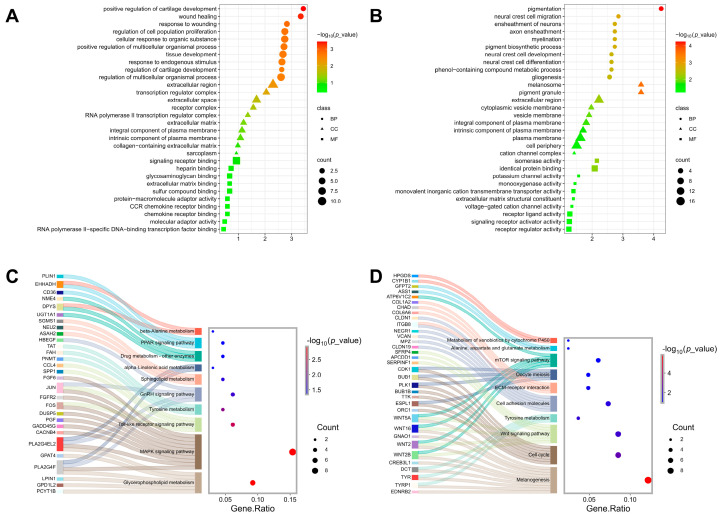
GO classification and KEGG pathway enrichment analysis of pectoralis muscle DEGs stratified by upregulated/downregulated expression patterns in Silkie chicken. Top 10 GO terms (BP, CC and MF) of downregulated (**A**) and upregulated (**B**) DEGs. Top 10 KEGG enrichment pathways of downregulated (**C**) and upregulated (**D**) DEGs.

GO enrichment analysis (biological processes) for downregulated DEGs in SK birds revealed significant enrichment in pathways related to positive regulation of cartilage development, tissue development, wound healing, cell proliferation, and response to injury (*p* < 0.05; Figure 2A). KEGG analysis further highlighted that the downregulated genes may be involved in glycerophospholipid metabolism, MAPK signaling, Toll-like receptor signaling, tyrosine metabolism, and GnRH signaling (*p* < 0.05; Figure 2C). All data are available in Supplementary Table S4.

Conversely, upregulated DEGs in SK birds showed pronounced enrichment in biological processes associated with melanin biosynthesis and deposition, including pigmentation, neural crest cell migration, and myelination processes (*p* < 0.05; Figure 2B). Cellular component analysis confirmed the enrichment in melanosome and pigment granule-related terms (*p* < 0.05; Figure 2B). KEGG pathway analysis reinforced these findings, identifying significant upregulation in melanogenesis, cell cycle regulation, WNT signaling pathways, and tyrosine metabolism (*p* < 0.05; Figure 2D). Collectively, these results suggest coordinated activation of melanogenic and WNT-related pathways in regulating ectopic melanin deposition in pectoralis muscle of Silkie chicken. Key genes driving these processes included *TYRP1*, *TYR*, *DCT*, and so on. All data are available in Supplementary Table S4.

Among the upregulated DEGs in SK birds, several genes showed strikingly high expression in pectoralis muscle of Silkie chickens (log2FC > 3, FDR < 0.05; Table 1). Notably, genes such as *TYRP1*, *MLANA*, *TYR*, *SLC6A15*, and *SLC38A11* were nearly exclusively expressed in Silkies, with minimal to undetectable levels in AA broilers (Table 1). In addition, other genes have established roles in melanin biosynthesis, such as *MLPH*, *EDNRB2*, *DCT*, *OCA2*, *PMEL*, *GPNMB*, *SLC24A5*, *TRPM1*, *SLC45A2*, *SOX10* (Table 1). All data are available in Supplementary Table S3.

### 3.2. Prediction of Genes Related to Melanin Deposition in the Pectoralis Muscle of Silkie

**Figure 3:**
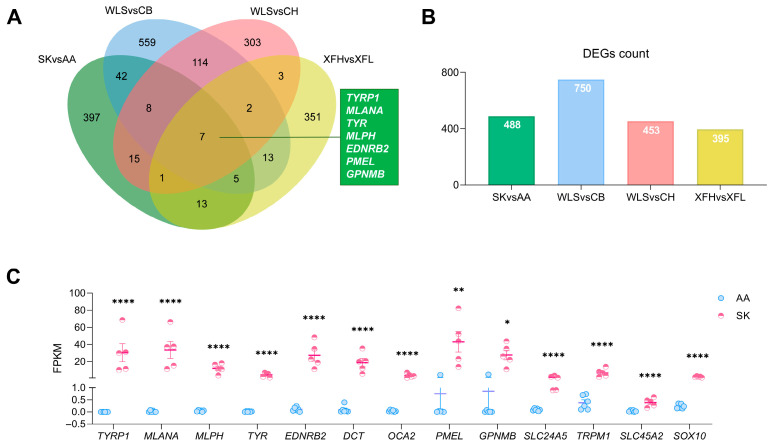
The information about 13 key melanogenic genes. (**A**) Venn analysis to screen for common pectoralis DEGs across multiple comparisons of dark- and light-breasted birds, including SK vs. AA comparison in this study, and Wuliangshan black-boned chicken (WLS) vs. Cobb broiler (CB) [19], WLS vs. Chahua chicken (CH) [19], and hyperpigmented Xuefeng black-bone chicken (XFH) vs. hypopigmented Xuefeng black-bone chicken (XFL) [18]. (**B**) DEG number for different comparisons. DEG screening criteria: SK vs. AA, WLS vs. CB, WLS vs. CH (|log2FC| ≥ 1, FDR < 0.05); XFH vs. XFL (|log2FC| ≥ 1, *p*-value < 0.05). (**C**) The expression levels of 13 key melanogenic genes. The data are presented as the mean ± SEM. FPKM, fragments per kilobase of transcript per million mapped reads. *, *p* < 0.05; **, *p* < 0.01; ****, *p* < 0.0001.

To identify key melanogenic genes governing melanogenesis in avian pectoralis muscle, the dataset from SK vs. AA in this study was integrated with published transcriptomes from other dark-pectoralis breeds (WLS and XFH) and non-pigmented controls (CB, CH, XFL and AA broilers). Venn analysis across these datasets pinpointed seven conservative melanogenesis genes (*TYRP1*, *MLANA*, *TYR*, *MLPH*, *EDNRB2*, *PMEL*, and *GPNMB*), which were highly expressed in SK chickens with log2FC > 3, FDR < 0.05 (Figure 3C and Table 1 and Table S3) and consistently upregulated across all dark-pectoralis breeds (Figure 3A,B and Supplementary Table S5). In addition, 6 other highly expressed genes with established roles in melanin biosynthesis (Figure 3C and Table 1), including *DCT* [37], *OCA2* [38], *SLC24A5* [42], *TRPM1* [43], *SLC45A2* [41,44], and *SOX10* [45], were also determined as key melanogenesis genes. Finally, 13 genes were defined as key melanogenesis regulators (Figure 3C and Table 1) and used for subsequent co-expression network analysis.

**Figure 4:**
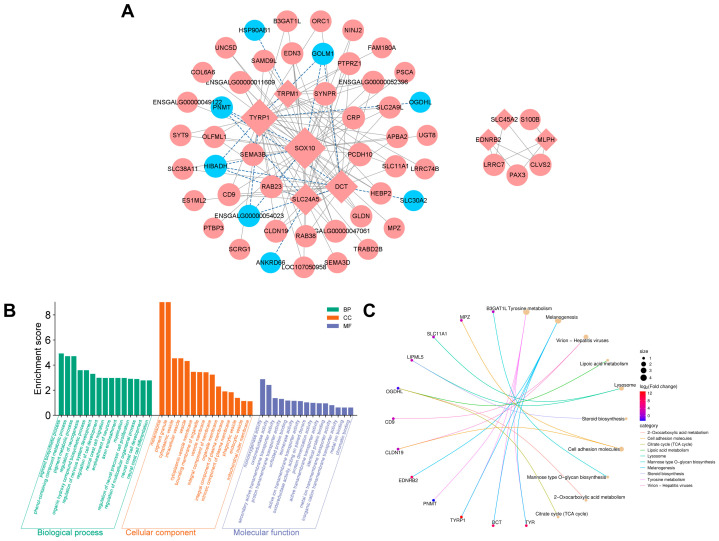
Construction of network interaction and GO/KEGG enrichment with identified high-confidence melanogenic regulators. (**A**) Construction of a network interaction map based on the high-confidence melanin-related DEGs (|*r*| > 0.9, *p* < 0.05) through the co-expression analysis with key melanogenic genes and DEGs. Blue circles indicate downregulated DEGs, red circles upregulated DEGs, red diamonds key melanogenic genes. Gray solid lines represent a positive correlation, and dashed blue lines represent a negative correlation. Top 15 GO terms in each GO category (BP, CC, MF) (**B**), and top 10 KEGG pathway enrichment (**C**) based on identified high-confidence melanin-related DEGs.

A high-confidence melanogenic network consisting of 67 genes was identified through co-expression network analysis (|*r*| > 0.9, *p* < 0.05) of 13 key melanogenic genes and DEGs between SK and AA (Figure 4A and Supplementary Table S6). This co-expression network analysis identified two independent melanocyte regulatory subnetwork modules. One larger module contains 60 high-confidence candidate regulators, with core hub genes including *SOX10*, *TYRP1*, *DCT*, *SLC24A5*, *TRPM1*, *SYNPR*, *CRP*, *PCDH10*, *RAB23*, and *SEMA3B*. This larger module, primarily represented by genes such as *SOX10*, *TYRP1*, and *DCT*, focuses on the catalytic processes of melanin synthesis and the supply of related raw materials. Meanwhile, the analysis also revealed a smaller subnetwork comprising *SLC45A2*, *EDNRB2*, *MLPH*, *S100B*, *PAX3*, *LRRC7*, and *CLVS2*. This smaller module, represented by *MLPH* and *SLC45A2*, emphasizes melanosome maturation, transportation, and intracellular distribution of pigment. Strikingly, several genes such as *PNMT*, *HIBADH*, *ANKRD66*, and *SLC30A2* showed strongly negative correlations with melanogenic core genes, positioning them as potential inhibitors of melanin deposition (Figure 4A and Supplementary Table S6).

GO functional enrichment analysis of these melanin-related regulators revealed significant associations with melanin biosynthesis (pigment biosynthesis and phenol-containing compound metabolism) and neural crest development (differentiation, migration, and myelination). Cellular component enrichment localized key genes to melanosomes (*TYR*, *PMEL*, *DCT*, *TYRP1*, and *HSP90AB1*) and membrane trafficking structures (*SLC11A1* and *SOX10*), underscoring their roles in melanosome biogenesis and dynamics. Molecular function analysis emphasized oxidoreductase activity (*TYR*, *DCT*, and *TYRP1*), transmembrane transport (*SLC11A1*), and monooxygenase activity, all critical for melanin synthesis and melanosome homeostasis (Figure 4B and Supplementary Table S6).

KEGG pathway enrichment identified tyrosine metabolism and melanogenesis as central hubs, driven by *TYR*, *DCT*, *TYRP1*, and *EDNRB2*. Notably, *PNMT*, a gene linked to catecholamine synthesis, was enriched in tyrosine metabolism. In addition, several other pathways were enriched, including virion-hepatitis viruses, lipoic acid metabolism, lysosome, and steroid biosynthesis (Figure 4C and Supplementary Table S6).

### 3.3. Metabolite Profiling of Chicken Pectoralis Muscle

**Figure 5:**
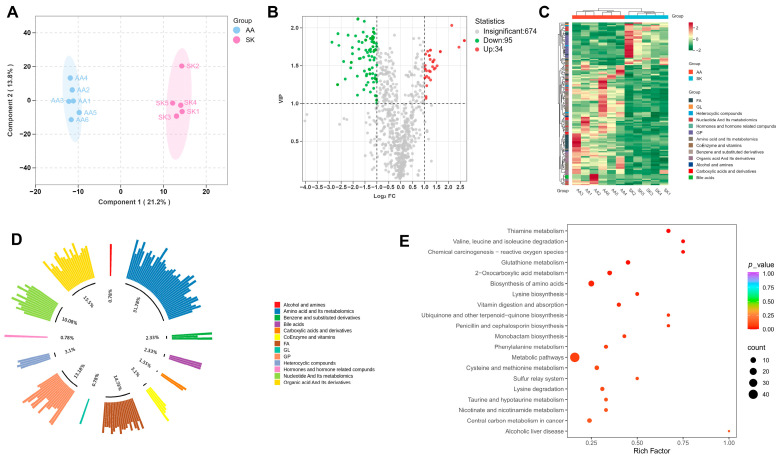
Differentially abundant metabolites (DAMs) analysis based on broad-targeted metabolomics in pectoralis muscle of SK vs. AA. (**A**) OPLS-DA analysis (the ellipse represents the 95% confidence intervals of the samples in each group), (**B**) Volcano plot, and (**C**) Heatmap of DAMs in SK vs. AA comparison. (**D**) Classification of DAMs based on first-level classification of substances. (**E**) Enriched KEGG metabolic pathway based on all pectoralis DAMs. Metabolites with VIP > 1 and |log2FC| ≥ 1 were considered as DAMs.

Beyond genetic regulation of melanin deposition, numerous studies have linked metabolites to the modulation of melanin accumulation. To further explore how metabolites in pectoralis muscle were regulated in dark-pectoralis chickens, broad-targeted metabolomics was performed through the comparison of SK vs. AA. The OPLS-DA revealed clear inter-group separation, with biological replicates clustering tightly within each group (Figure 5A). The correlation scatter plot demonstrates high reliability of the metabolomics data (Supplementary Figure S1B). Based on stringent thresholds (VIP > 1, |log2FC| ≥ 1), this analysis yielded 34 upregulated and 95 downregulated metabolites in Silkie chickens (Figure 5B and Supplementary Table S7). Cluster analysis of DAMs revealed a clear distinction between two breeds (Figure 5C). These DAMs mainly belong to amino acid and its metabolomics (31.78%) (Figure 5D), and further subclassification revealed that they consisted of amino acid derivatives (53.6%), small peptides (24.3%), and amino acids (21.9%) (Supplementary Table S7). KEGG enrichment analysis revealed that these DAMs in SK vs. AA were enriched in thiamine metabolism, chemical carcinogenesis-reactive oxygen species, valine, leucine, and isoleucine degradation, glutathione metabolism, 2-oxocarboxylic acid metabolism, amino acid biosynthesis, and lysine biosynthesis (Figure 5E and Supplementary Table S7).

### 3.4. Joint Analysis of Transcriptomics and Metabolomics in the Pectoralis Muscle

**Figure 6:**
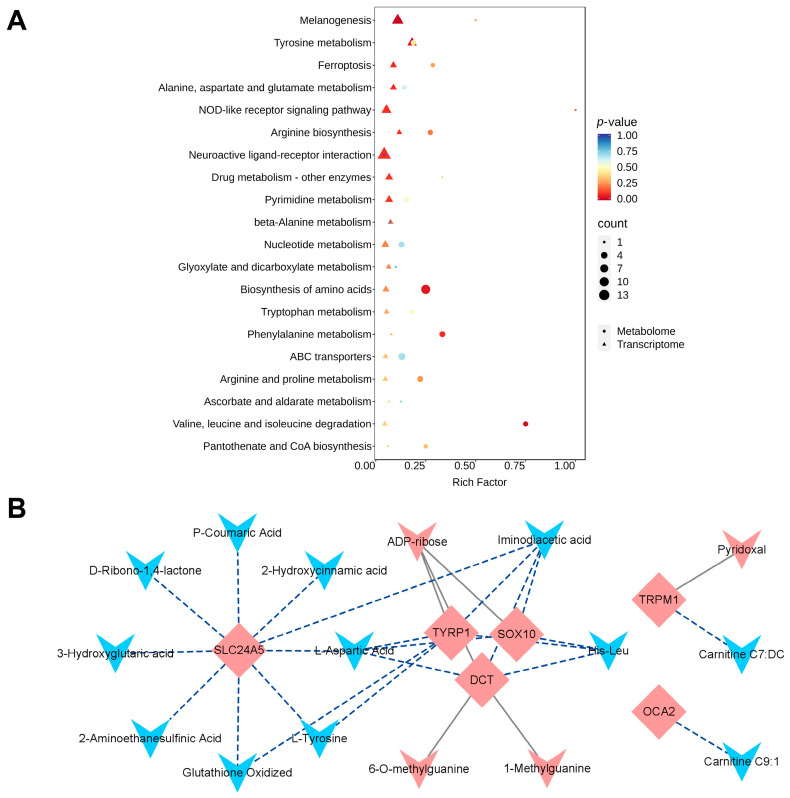
Joint analysis of transcriptomics and metabolomics in the pectoralis muscle. (**A**) Information on KEGG pathways co-enriched with DEGs and DAMs. (**B**) Construction of network interaction map based on the key melanogenic genes and all DAMs. Blue triangles downregulated represent DAMs, red triangles upregulated DAMs, and red diamonds key core genes. Gray solid lines represent a positive correlation; dashed blue lines represent a negative correlation.

Transcriptomic data of pectoralis muscle were further integrated with metabolomic data for a joint KEGG analysis. This combined approach revealed the common enrichment in the melanogenesis and tyrosine metabolism pathways, which are known to influence melanin deposition. Additionally, various amino acid metabolism pathways, including those for alanine, aspartate, glutamate, arginine, tryptophan, phenylalanine, and glutathione, were also commonly enriched (Figure 6A and Supplementary Table S8).

To identify the potential metabolites associated with melanin deposition, an interaction analysis was performed between 13 key melanin regulatory genes (Table 1), and all DAMs. The analysis showed that metabolites, such as ADP-ribose, 6-O-methylguanine, 1-methylguanine, and pyridoxal (belonging to top 10 upregulated metabolites, Table 2), were positively correlated with the expression of key melanogenic genes. Conversely, metabolites such as iminodiacetic acid, L-aspartic acid, His-Leu, L-tyrosine, p-coumaric acid, 2-aminoethanesulfinic acid, glutathione oxidized, and 2-hydroxycinnamic acid (belonging to top 10 downregulated metabolites, Table 2) displayed negative correlation with genes involved in melanin synthesis (Figure 6B and Supplementary Table S8).

## 4. Discussion

The main objective of this study was to unravel the genetic and metabolic mechanisms underlying ectopic melanin deposition in the pectoralis muscle of SK chickens, leveraging the extreme phenotypic contrast between SK chickens (with extensive muscle melanin deposition) and AA broilers (without muscle melanin deposition). It aimed to systematically identify DEGs, DAMs, and key regulatory pathways involved in muscle melanogenesis, construct gene-metabolite interaction networks through integrated transcriptomic and metabolomic analyses, and pinpoint potential molecular markers and regulatory factors for the genetic improvement of melanin-related traits in poultry. The distinctive dark pigmentation of SK chicken pectoralis muscle, which is in extreme contrast to the melanin-free phenotype of AA broilers, offers a unique model to dissect the genetic and metabolic underpinnings of ectopic melanin deposition in skeletal muscle. The integrated transcriptomic and metabolomic analyses reveal a coordinated interplay between melanogenic pathways, regulatory networks, and metabolic adaptations driving this phenotype. Here, the strand-specific RNA sequencing was employed to identify mRNA genes influencing melanin deposition. Compared to conventional RNA-Seq, this technique offers critical insights into the origin of transcript strands, facilitating precise gene assignment and accurate quantification, particularly for overlapping genes and closely positioned antisense transcripts [46]. Below, these findings were contextualized within the broader understanding of melanogenesis, highlighting novel insights into its regulation in avian muscle tissue.

### 4.1. Transcriptomic Signatures of Melanogenesis

The upregulation of melanogenic genes (such as *TYRP1*, *TYR*, *DCT*, and *EDNRB2*) and pathways (melanogenesis, tyrosine metabolism and WNT signaling) in SK chickens aligns with their established roles in melanocyte differentiation, melanosome biogenesis, and pigment synthesis [22,29,36,47], indicating the functional crosstalk of WNT signaling and tyrosine metabolism in muscle melanin deposition [48,49,50,51,52]. Notably, *TYRP1* exhibited an extraordinary log2FC of 12.91 (Table 1), while *TYR* and *EDNRB2* also showed extreme expression divergence, which validates their role as key drivers of muscle pigmentation. Critically, Venn analysis, integrating transcriptomic data from multiple dark- and light-breasted chicken populations, confirmed that seven core genes (*TYRP1*, *MLANA*, *TYR*, *MLPH*, *EDNRB2*, *PMEL*, and *GPNMB*) were consistently upregulated across all dark-pectoralis breeds (Figure 3). This cross-population conservation strongly supports that these signals reflect pigmentation-specific regulation rather than genetic background effects, thereby directly addressing concerns about the choice of AA chickens as controls.

Co-expression network analysis identified *SOX10* and *SLC45A2* as hub regulators, consistent with their known functions in melanocyte survival and ion transport [45,53,54]. Intriguingly, beyond these known regulatory factors, this study uncovered novel regulatory candidates: *PNMT* (a catecholamine biosynthesis enzyme) [55] and *HIBADH* were strongly negatively correlated with core melanogenic genes, suggesting they may act as checkpoints to constrain excessive pigment deposition. *PNMT*’s enrichment in tyrosine metabolism further implies crosstalk between neurotransmitter synthesis and melanin biosynthesis [56,57,58], expanding the current understanding of the systemic regulation of melanin deposition.

Notably, downregulated genes in SK chickens were associated with cartilage development, cell proliferation, and wound healing. This suggests a trade-off between muscle growth and melanin deposition [59], as the suppression of proliferative pathways may redirect cellular resources toward pigment synthesis. KEGG analysis linking these genes to glycerophospholipid metabolism and MAPK signaling implies potential crosstalk between lipid homeostasis and melanogenesis [54], though further validation is needed.

### 4.2. Metabolomic Insights into Melanin Substrate Dynamics

Metabolomic profiling identified 129 differentially abundant metabolites between SK and AA chickens. A key paradox emerged: despite robust upregulation of melanogenic genes, L-tyrosine, a pivotal melanin precursor [60], and its derivatives (L-tyrosine methyl ester, N-acetyl-L-tyrosine, Ala-Tyr) were significantly depleted in SK muscle (Table 3). This depletion suggests intense metabolic flux toward pigment synthesis, where heightened tyrosinase activity, potentially driven by *TYR*, *TYRP1*, and *DCT* upregulation, rapidly consumes local tyrosine pools. This may provide direct metabolomic evidence for the substrate exhaustion hypothesis underlying dark meat formation.

Concomitant downregulation of oxidized glutathione (an important antioxidant) in SK muscle suggests that melanin synthesis induces a pro-oxidative microenvironment [61,62]. This aligns with the well-documented role of glutathione in regulating eumelanin/pheomelanin ratios [24] and its skin-whitening effects in humans [63,64,65,66]. Additionally, p-coumaric acid, a known competitive inhibitor of tyrosinase [67,68,69,70], was downregulated in SK chickens, potentially representing a compensatory mechanism to fine-tune tyrosinase activity and prevent unchecked pigment deposition. Taurine could suppress tyrosinase activity and reduce melanin synthesis [71,72].

Notably, ADP-ribose and pyridoxal (top upregulated DAMs) were positively correlated with core melanogenic genes, suggesting the potential function of these metabolites on melanin deposition. Previous studies have demonstrated that pyridoxal modulates melanin synthesis in melanoma cells [73]. ADP-ribose is known to activate TRPM channels linked to melanin content [43,74,75] and regulate melanoma cell differentiation [76]. In this study, ADP-ribose shows a strong correlation with *TRPM1* (*r* = 0.8, Supplementary Table S8), a TRPM family member involved in muscle melanin deposition [17]. This suggests that ADP-ribose may mediate melanin production in chicken pectoralis muscle via TRPM channels.

### 4.3. Integrated Pathways and Systemic Regulation

Integrated multi-omics analysis identified melanogenesis and tyrosine metabolism as central hubs bridging transcriptional and metabolic perturbations. The disconnect between upregulated melanogenic genes and depleted L-tyrosine derivatives underscores the importance of post-transcriptional and metabolic regulation, likely via feedback inhibition or compensatory mechanisms to balance pigment synthesis with cellular homeostasis. Enrichment of glutathione and phenylalanine metabolism pathways further highlights the extensive metabolic rewiring required to sustain melanin production, potentially at the cost of redox balance or energy storage [77].

### 4.4. Limitations and Future Directions

While this study leverages extreme phenotypic contrast to efficiently capture core regulatory signals, it has some limitations. First, the focus on pectoralis muscle limits the understanding of systemic melanin regulation; future multi-tissue analyses should delineate the local and systemic control of pigment deposition. Second, despite cross-population validation, the use of AA chickens as controls cannot fully eliminate genetic background effects. Follow-up studies using SK chicken populations with variable melanin deposition (or F2 hybrids) will further validate candidate regulators. Third, regarding sample size, one low-quality SK sample was excluded from transcriptome analysis (initial *n* = 6 per group), resulting in an effective sample size of 5 for the SK group. This may have compromised transcriptomic statistical power to some extent, mitigated by stringent statistical thresholds and cross-validation of key findings with metabolomic data and published literature. Fourth, the constructed functional network has inherent limitations and could be further improved by advancing network construction (e.g., establishing higher-order regulatory networks via machine learning). Fourth, this study identifies novel candidates, such as *PNMT*, *HIBADH*, *ANKRD66*, and *SLC30A2*, as potential suppressors of melanogenesis. Functional validation of these novel candidates via in vitro (melanocyte culture, gene silencing/overexpression) and in vivo (transgenic models) assays is needed to confirm their roles in muscle melanogenesis.

## 5. Conclusions

By integrating transcriptomic and metabolomic profiling of chickens with extreme muscle melanin phenotypes, we identified a core gene-metabolite network governing ectopic melanin deposition that is characterized by tyrosine pool exhaustion and redox imbalance. The cross-population validation confirms that these signals are pigmentation-specific, addressing concerns about genetic background effects. The discovery of novel regulators such as *PNMT* and *HIBADH* expands the current understanding of melanogenesis, while the identified core molecules including *TYRP1*, *TYR*, L-tyrosine, and oxidized glutathione provide valuable molecular markers for the genetic improvement of melanin-related traits in poultry. This work highlights the power of extreme phenotypic contrast in dissecting complex traits and offers a framework for bridging genetic and metabolic mechanisms in livestock research.

## Figures and Tables

**Table 1:** Highly expressed DEGs in pectoralis of Silkie chickens relative to AA broilers (log2FC > 3 and FDR < 0.05).

Gene Name	AA (FPKM)	SK (FPKM)	log2FC	FDR	Supported by Other Published RNA-Seq Data	Related Functional Reports	Key Melanogenic Genes
*TYRP1*	0.00	30.43	12.91	****	WLS vs. CB; WLS vs. CH; XFH vs. XFL	[29,30]	√
*MLANA*	0.01	33.63	10.50	****	WLS vs. CB; WLS vs. CH; XFH vs. XFL	[31]	√
*MLPH*	0.04	12.18	8.13	****	WLS vs. CB; WLS vs. CH; XFH vs. XFL	[32,33]	√
*TYR*	0.02	4.45	8.01	****	WLS vs. CB; WLS vs. CH; XFH vs. XFL	[34]	√
*EDNRB2*	0.08	27.32	7.86	****	WLS vs. CB; WLS vs. CH; XFH vs. XFL	[35,36]	√
*SLC6A15*	0.02	4.39	7.82	****	XFH vs. XFL	—	
*DCT*	0.09	18.79	7.53	****	WLS vs. CB; XFH vs. XFL	[37]	√
*SLC38A11*	0.00	0.65	7.23	****	—	—	
*OCA2*	0.03	3.72	6.50	****	—	[38]	√
*CRP*	3.72	320.12	6.40	****	XFH vs. XFL	—	
*PMEL*	0.75	43.08	5.92	**	WLS vs. CB; WLS vs. CH; XFH vs. XFL	[39]	√
*GPNMB*	0.85	27.75	5.11	*	WLS vs. CB; WLS vs. CH; XFH vs. XFL	[40,41]	√
*TUBB3*	0.05	1.29	4.34	****	WLS vs. CH; XFH vs. XFL	—	
*SLC24A5*	0.09	1.84	4.20	****	XFH vs. XFL	[42]	√
*TRPM1*	0.31	6.99	4.09	****	—	[43]	√
*SYNPR*	0.36	6.52	3.89	****	WLS vs. CB;	—	
*EOMES*	0.04	0.82	3.63	***	—	—	
*PSCA*	0.38	7.17	3.62	****	WLS vs. CB; WLS vs. CH	—	
*PCDH10*	0.03	0.46	3.58	****	—	—	
*SLC2A9L*	0.13	1.68	3.44	****	—	—	
*SLC45A2*	0.03	0.39	3.40	****	—	[41,44]	√
*SOX10*	0.19	2.28	3.21	****	WLS vs. CB	[45]	√

Note: SK, Silkie chickens; AA, Arbor Acres broilers; Wuliangshan black-boned chicken (WLS) vs. Cobb broiler (CB) [19], WLS vs. Chahua chicken (CH) [19], and hyperpigmented Xuefeng black-bone chicken (XFH) vs. hypopigmented Xuefeng black-bone chicken (XFL) [18]. FPKM, fragments per kilobase of transcript per million mapped reads. * means *p* < 0.05; ** means *p* < 0.01; *** means *p* < 0.001; **** means *p* < 0.0001. Identified key melanogenic genes are marked with “√”.

**Table 2:** Top 10 upregulated/downregulated DAMs in Silkie chickens (relative to AA broilers, sorted by VIP values).

Compounds	Class I	VIP	log2FC	Type
ADP-ribose	Nucleotide and its metabolomics	2.04	2.14	up
6-O-methylguanine	Nucleotide and its metabolomics	1.83	2.67	up
1-methylguanine	Nucleotide and its metabolomics	1.83	2.67	up
Pyridoxal	Heterocyclic compounds	1.75	2.44	up
LPE(16:0/0:0)	GP	1.71	1.25	up
LPE(0:0/16:0)	GP	1.71	1.25	up
LPE(14:0/0:0)	GP	1.69	1.13	up
LPE(0:0/22:6)	GP	1.69	1.66	up
LPE(22:6/0:0)	GP	1.69	1.66	up
LPC(0:0/18:2)	GP	1.65	1.05	up
Iminodiacetic acid	Organic acid and its derivatives	2.12	−1.79	down
L-aspartic acid	Amino acid and its metabolomics	2.12	−1.80	down
His-Leu	Amino acid and its metabolomics	2.09	−1.53	down
L-tyrosine	Amino acid and its metabolomics	1.99	−1.14	down
p-Coumaric acid	Benzene and substituted derivatives	1.95	−1.14	down
2-Hydroxycinnamic acid	Benzene and substituted derivatives	1.95	−1.14	down
Glutathione oxidized	Amino acid and its metabolomics	1.94	−1.57	down
Thymine	Nucleotide and its metabolomics	1.93	−1.10	down
Carnitine C7-OH	FA	1.92	−2.63	down
2-Aminoethanesulfinic acid	Organic acid and its derivatives	1.90	−2.21	down

Note: VIP, Variable Importance in Projection score.

**Table 3:** The information about the modulation of tyrosine and its related metabolites in Silkie chickens (relative to AA broilers).

Compounds	Class I	VIP	log2FC	Type
L-tyrosine	Amino acid and its metabolomics	1.99	−1.14	down
Ala-Tyr	Amino acid and its metabolomics	1.72	−1.76	down
N-Acetyl-L-tyrosine	Amino acid and its metabolomics	1.71	−1.46	down
L-tyrosine methyl ester	Amino acid and its metabolomics	1.10	−1.02	down

## Data Availability

RNA-seq data have been deposited in the SRA database under accession numbers PRJNA1039253 and PRJNA1039316.

## Supplementary Materials

The following supporting information can be downloaded at https://www.mdpi.com/article/10.3390/ani16020252/s1, Supplementary Figure S1. The Pearson correlation coefficient of samples for pectoralis muscle transcriptomics (A) and metabolomics (B). Supplementary Figure S2. Validation of the RNA-seq data using qRT-PCR. The Y-axis on the left represents the data from qRT-PCR, and the right Y-axis shows the FPKM values obtained from the RNA-seq data. The data are presented as the mean ± SEM. Supplementary Table S1. Primers for validation of pectoralis muscle transcriptomic data with qRT-PCR. Supplementary Table S2. Summary of transcriptomic data quality. Supplementary Table S3. Information about DEGs and highly expressed DEGs in SK vs. AA. Supplementary Table S4. Information about upregulated/downregulated DEGs in SK vs. AA for GO classification (Figure 2A,B) and KEGG pathway enrichment analysis (Figure 2C,D). Supplementary Table S5. Venn analysis among multiple comparisons of dark- and light-breasted birds (Figure 3A), including this SK vs. AA comparison, and Wuliangshan black-boned chicken (WLS) vs. Cobb broiler (CB) [19], WLS vs. Chahua chicken (CH) [19], and hyperpigmented Xuefeng black-bone chicken (XFH) vs. hypopigmented Xuefeng black-bone chicken (XFL). Supplementary Table S6. Co-expression network-based discovery of high-confidence melanogenic genes. Information about the correlation between key melanogenic genes and all DEGs in SK vs. AA, high-confidence melanogenic genes (|*r*| > 0.9, *p* < 0.05) identified via co-expression analysis (Figure 4A), GO classification (Figure 4B) and KEGG pathway enrichment (Figure 4C) of high-confidence melanogenic genes. Supplementary Table S7. Detailed information about all DAMs and KEGG pathway enrichment (Figure 5E) in SK vs. AA. Supplementary Table S8. Multi-omics analysis reveals gene-metabolite interactions in melanogenesis and potential melanin-related DAMs. Information about the co-enriched KEGG pathways of DEGs and DAMs in SK vs. AA (Figure 6A), correlations between key melanogenic genes and all DAMs, and DAMs (|*r*| > 0.9, *p* < 0.05) identified via co-expression analysis (Figure 6B).
